# 

**Published:** 1970-01

**Authors:** 


Bristol Medico-Chirurgical Journal Vol. 85 (i) No. 313

### CONTENTS

Editorial: Changes in the Journal ... ... ... ... ... ... 1

Vipers and Viper Bites in the West Country ... ... ... ... ... 3

**C. J. Moran**

Exchange Transfusion in Severely Anæmic Adults with Heart Failure ... ... ... ... ... ... ... ... ... ... 7

**A. P. Cole**

The Future Maternity Services of Bristol ... ... ... ... ... 9

**J. Sluglett**

Canynge Hall 1933-1969 ... ... ... ... ... ... ... 11

**R. C. Wofinden**

Ave atque Vale ... ... ... ... ... ... ... ... ... 17

**J. M.**

Southmead Orchestral Society ... ... ... ... ... ... 19

Wisdom from the Past ... ... ... ... ... ... ... ... 21

The Medical Library ... ... ... ... ... ... ... ... 23

Bristol Medico-Chirurgical Society ... ... ... ... ... ... 25

Postgraduate Education ... ... ... ... ... ... ... 26

South-West Orthopædic Club ... ... ... ... ... ... ... 27

Notes and News ... ... ... ... ... ... ... ... 29

Newly-appointed Consultants ... ... ... ... ... ... ... 30

Notices ... ... ... ... ... ... ... ... ... 30

F. Bailey & Son Ltd., Dursley, Glos.

